# The Spiky Side of Urban Wildlife, First Detection of a Zoonotic Assemblage of *Giardia duodenalis* in European Hedgehogs (*Erinaceus europaeus*) from Italy

**DOI:** 10.1007/s11686-025-01009-y

**Published:** 2025-03-26

**Authors:** Leonardo Brustenga, Giulia Rigamonti, Iolanda Moretta, Giulia Morganti, Valentina Calgaro, Giuseppe Giglia, Manuela Diaferia, Elvio Lepri, Livia Lucentini, Marco Lalle, Fabrizia Veronesi

**Affiliations:** 1 Department of Veterinary Medicine, University of Perugia, Via San Costanzo 4, Perugia, Italy; 2 WildUmbria Wildlife Rescue Center, Perugia, Italy; 3 Department of Chemistry, Biology and Biotechnology, University of Perugia, Via del Giochetto, Perugia, Italy; 4 Unit of Foodborne and Neglected Parasites, Department of Infectious Diseases, Istituto Superiore di Sanità, Rome, Italy

**Keywords:** *Giardia duodenalis*, *β*-giardin, AI-like zoonotic assemblage, European hedgehog, Italy

## Abstract

**Purpose of Review:**

*Giardia duodenalis* is a flagellate protozoan parasite of several mammals, that is transmitted via the fecal-oral route and causes gastrointestinal diarrheal illness. Molecular analyses of several genetic markers have classified *G. duodenalis* into eight assemblages (A to H) exhibiting different host specificities. Assemblages A and B have zoonotic potential and infect a broad range of hosts. The European hedgehog (*Erinaceus europaeus*) is one of the wild species well-adapted to live in close proximity to humans. Therefore, surveying potential zoonotic parasites harbored by hedgehogs can also be of public health concern, particularly in urban settings with high animal densities.

**Recent findings:**

Coproparasitological examinations are routinely conducted on hedgehogs admitted to WildUmbria Wildlife Rescue Center. *Giardia duodenalis* cysts were found in the fecal flotation of a hedgehog and were further tested using a direct immunofluorescent assay and PCR-RFLP molecular analysis of the *β*-giardin gene for assemblage and sub-assemblage characterization. The RFLP protocol attributed the samples to the AI-like zoonotic assemblage. Additionally, trophozoites attached to the intestinal mucosa of the small intestine were detected via histological techniques.

**Summary:**

Although *G. duodenalis* has been detected in hedgehogs from several countries, it was never before reported in Italian hedgehogs. The identification of a zoonotic assemblage in hedgehogs suggests the potential for the parasite to be shared between wild and domestic environments, with pets possibly serving as bringing hosts.

The genus *Giardia* comprises flagellate protozoans inhabiting the small intestine of a wide range of vertebrate hosts including humans [1]. *Giardia* has a two-stages lifecycle comprising: the trophozoite, the motile vegetative form that attaches to the enterocytes in the small intestine using a ventral disk; and the cyst, the infective form of the parasite, which is excreted with feces and can survive in the environment [1, 2]. Among the eight species of *Giardia* known to date, *G. duodenalis* is not only the most studied and widespread, but also the only species known to infect humans [1]. DNA [3, 4] and isozyme analysis [5] indicate that *G. duodenalis* is as a complex of eight morphologically identical but genetically different groups, also known as assemblages (A to H), each displaying different host specificity [6]. Assemblages A and B have shown a degree of zoonotic potential, as they can infect a broad range of mammalian hosts. Recent finding show that the biodiversity of *G. duodenalis* is particularly complex and that multilocus sequence typing (MLST) of several loci like the genes for *β*-giardin (*bg*), triosophosphate isomerase (*tpi*) and glutamate dehydrogenase (*gdh*) allowed the further subclassification of assemblages A and B into sub-assemblages (AI, AII, AIII, BIII and BIV) [3, 6]. Assemblage A is of particular concern for human health; while sub-assemblage AII is primarily found in humans, sub-assemblage AI is mostly found in animals but has also zoonotic potential [7, 8].

Greenspaces in urban environments like public parks and private gardens can be favorable for a wide range of vertebrate fauna [9]. One of the most successful urban-adapted mammals is the European hedgehog (*Erinaceus europaeus*), a small, nocturnal insectivore that is commonly found in the above-mentioned settings in both small villages and large cities [10]. Considering the wide variety of pathogens that hedgehogs can harbor [11, 12], and the increasing overlap of their habitats with human settlements, assessing the presence of zoonotic pathogens in hedgehog populations is of public health interest. For this reason, hedgehogs rescued by WildUmbria wildlife rescue center (WRC) in Central Italy, are routinely examined to assess the presence of gastrointestinal and bronchopulmonary parasites.

An adult hedgehog female was rescued at the end of 2023 from a private garden in the suburban area of Perugia (Central Italy). The animal was active during the day but was in poor body condition. Therefore, it was admitted for first medical care at the Veterinary Teaching Hospital (OVUD) of the Department of Veterinary Medicine, University of Perugia. It was individually housed in a cage from the bottom of which faecal samples were collected before the administration of any anti-parasitic or antibiotic treatment and maintained at 4 °C until processing. Fecal flotations were carried out in Sheather’s solution (specific gravity 1.25) and in 33% ZnSO_4_ solution (specific gravity 1.18) and inspected after 20 min. Fresh feces were also subjected to a direct fluorescent assay using Merifluor (Meridian Bioscience, Cincinnati, Ohio), designed for the simultaneous detection of *Cryptosporidium* and *Giardia* antigens, following the manufacturer’s instructions.

DNA extraction from feces was carried out using the QIAamp Fast DNA Stool Mini Kit (QIAGEN, Hilden, Germany), following the manufacturer’s instructions for pathogen detection. PCR amplification targeting a 511 bp fragment of the *β*-giardin (*bg*) gene was performed using a nested-PCR protocol as previously described [13]. All PCR reactions were performed on a final volume of 25 µL as follows: 12.5 µL of BlasTaq 2x PCR MasterMix (Applied Biological Materials, Vancouver, Canada), 1 µL of each of the 10 µM forward and reverse primers, 2 µL of template DNA and molecular biology grade nuclease-free water (Promega, Madison, Wisconsin) to final volume. Negative controls using nuclease-free water and positive controls using in-house, already genotyped, *G. duodenalis* isolates were included in each round of amplification. Amplicon quality and product size were checked in SafeView (Applied Biological Materials, Vancouver, Canada) stained 2% agarose gel by comparison with a 100 bp DNA ladder marker (Applied Biological Materials, Vancouver, Canada). Amplicons of the expected size were purified using ExoSAP-IT PCR Product Cleanup (Thermo Fisher Scientific, Waltham, Massachusetts) outsourced for bidirectional sequencing to Eurofins Genomics (https://eurofinsgenomics.eu) using the inner primer set.

All sequencing data were processed with MEGA v.11 software [14]. Electropherograms were visually inspected to rule out the presence of heterozygous sites [15,16] that could interfere with the functioning of restriction enzymes. The sequences obtained were taxonomically attributed using the nucleotide BLAST algorithm (https://blast.ncbi.nlm.nih.gov/Blast.cgi) and subsequently used to perform an in-silico RFLP protocol with the online software NEBcutter 3.0 (https://nc3.neb.com/NEBcutter), provided by New England BioLabs Inc., using the restriction enzyme *Hae*III [GG/CC] [13] to identify *G. duodenalis* assemblages and sub-assemblages. MEGA v.11 was also used to confirm that the protein translation of the obtained sequence did not alter the amino acid sequence or potentially the protein structure in the presence of mutations.

The collected feces presented as formed, soft, of typical coloration and not diarrohic, in spite of the subcutaneous administration of 10 mg/kg enrofloxacin (Baytril ^®^) the animal died spontaneously three days after the rescue and a full necropsy was performed. During the gross examination, samples of liver, lung, spleen, kidney and small intestine were collected and fixed in 10% buffered formalin to be submitted for histology, sections were cut at a thickness of 3 μm and stained with Hematoxylin-Eosin. Along with *G. duodenalis* cysts (Fig. 1A), the coproparasitological analysis showed the presence of L_1_ larvae of the lungworm *Crenosoma striatum*, eggs of the lungworm *Eucoleus aerophilus* and of the intestinal fluke *Brachylaemus erinacei.* The presence of cysts of *G. duodenalis* was further confirmed by the Merifluor fluorescent assay that also excluded the presence of *Cryptosporidium* spp. (Fig. 1B).

Macroscopic examination of the animal revealed moderate and bilateral multifocal granulomatous pneumonia and severe, locally extensive suppurative bronchopneumonia. Bundles of adult *C. striatum* were observed in the lumen of bronchi and bronchioles, possibly exacerbating the clinical symptoms of pneumonia. Histologically, the lung exhibited adult nematodes in the lumen of bronchioles, accompanied by a mild to moderate mixed inflammatory infiltrate predominantly composed of mononuclearFig. s (Fig. 1C). The pulmonary *interstitium* was diffusely and moderately expanded by mononuclear cells, with multiple foci of macrophages, epithelioid cells and rare multinucleated giant cells surrounding larval nematodes, indicating granulomatous pneumonia. The cranioventral lobes presented areas severely affected by necrosis and suppuration with involvement of the airways and surrounding parenchyma (bronchopneumonia; not shown), likely the cause of death. Macroscopic examinations of the small intestine revealed the presence of catarrhal material. Histologically, the villar lamina propria was mildly expanded by the presence of lymphocytes and plasma cells. The epithelium showed an increased number of muciparous cells with presence of numerous *G. duodenalis* trophoFig. s (Fig. 1D) on its luminal side.

**Fig. 1:**
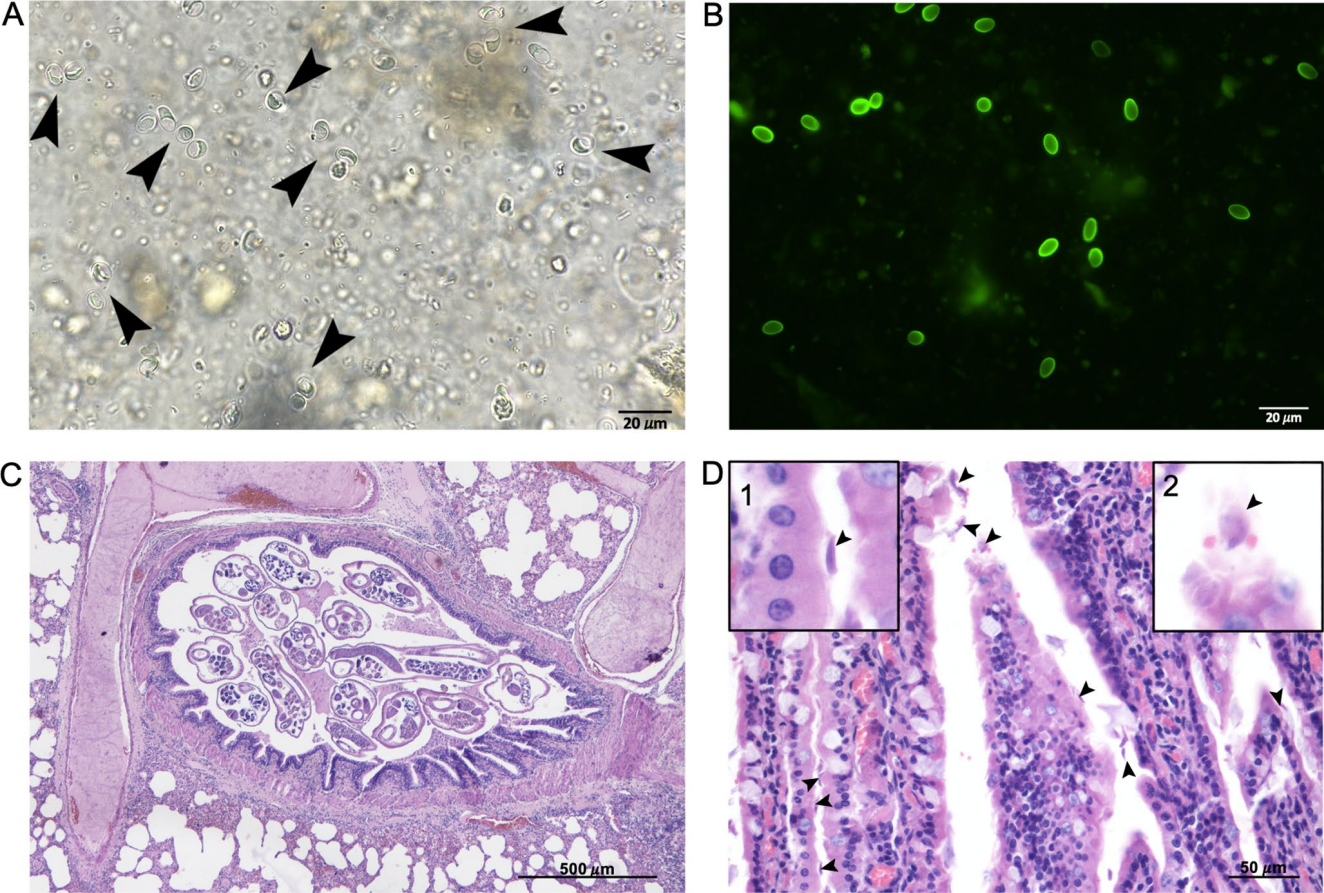
(**A**) *Giardia duodenalis* cysts, Fecal flotation in Sheather solution, brightfield microscopy, 40x magnification; (**B**) *Giardia duodenalis* cysts, Merifluor assay, direct fluorescence, 40x magnification; (**C**) Lung: cross-section of adult nematodes in the lumen of a bronchiole (x40). (**D**) Jejunum: *Giardia duodenalis* trophozoites (arrowheads) overlaying the intestinal epithelium, with scant lymphoplasmacytic infiltrate in the lamina propria (x400); the two insets show higher magnifications of: a trophozoite attached to the apical surface of an enterocyte (inset 1) and a trophozoite in which are visible the two nuclei (inset 2)

The DNA sequence produced was deposited in GenBank with the accession number PP960564. The nBLAST analyses matched the amplified sample with the *β*-giardin gene of *G. duodenalis* with 99.8% homology. The in silico RFLP protocol produced three fragments of 351 bp, 110 bp and 50 bp (Fig. 2A). Although the restriction pattern did not match any of the known restriction patterns for the seven known assemblages, the analysis of the polymorphisms, according to Lalle et al. [13], attributed the isolate to the sub-assemblage AI. This discrepancy is due to a polymorphic site at position 313, where a cytosine-to-thymine substitution prevents the restriction enzyme from digesting the DNA (Fig. 2B), resulting in the fragmentation of the 351 bp in two fragments of 201 bp and 150 bp.

The visual analysis of the electropherogram allowed to rule out the possibility of contamination, heterozygosity or of the contemporary presence of a mixed infection by parasites of different assemblages [17], as there were no underlying base-calls throughout the electropherograms and both forward and reverse sequences exhibited the same polymorphism. Additionally, the same polymorphism was found in another sequence deposited in GenBank (accession number LC341569) [18], which was amplified from cat stool sample positive for *G. duodenalis* in Japan and also attributed to assemblage AI. This polymorphism has been detected in a previously established isolate representing an assemblage AI-type (KO188) derived from a G. *duodenalis* infected cat [19]. The amino acid translation showed no changes since the mutation sites produces two codons: GCC, typical of the AI sub-assemblage, and GCT, detected in the AI-like sub-assemblage, both encoding for alanine. The amino acid sequence obtained showed 100% homology with the deposited sequences for *G. duodenalis β*-giardin protein. Therefore, the mutation found in the *G. duodenalis* isolate from the European hedgehog is a silent mutation that does not alter the structure or function of the translated protein in the parasite’s physiology.

**Fig. 2:**
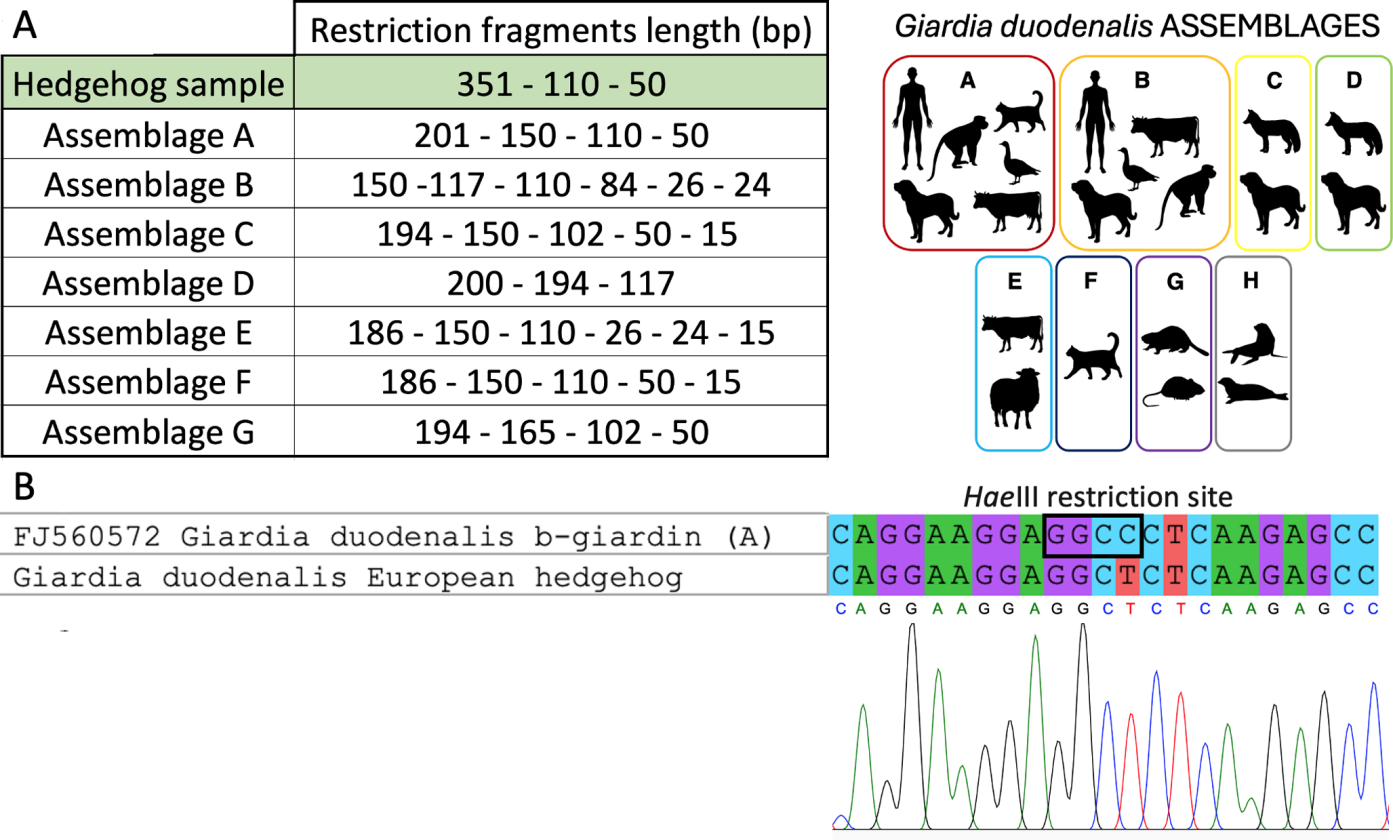
**A**. Restriction patterns obtained from the PCR-RFLP protocol applied to the hedgehog sample (green row) and to the 8 *G. duodenalis* assemblages [13]; **B**. Comparison of the *Hae*III restriction site in a *G. duodenalis* (assemblage A) isolate and on the sample from the European hedgehog. As evident from the electropherogram the C → T mutation is a valid polymorphism

The substantial parasitic burden observed in European hedgehogs can be explained by their behavioral and dietary habits, in fact a significant portion of their diet consists of preys such as earthworms and gastropods, which serve as intermediate or paratenic hosts for numerous parasites [11,12]. While *C. striatum* and *B. erinacei* are species-specific parasites, the other parasites detected have a zoonotic potential and can pose threats to both companion animals and humans. For example, *E. aerophilus*, a cosmopolitan lungworm, causes lung capillariosis in various carnivores and insectivores, with the red fox (*Vulpes vulpes*) being its primary host and reservoir [20]. Infestations of *E. aerophilus* in cats and dogs can lead to moderate to severe respiratory symptoms, including nasal discharge, coughing, dyspnea, bronchitis, and pneumonia [21]. The zoonotic potential of *E. aerophilus* is supported by occasional cases of lung capillariasis in humans [22]. It is also speculated that human lung capillariasis may be widely underreported, as most of the cases were diagnosed incidentally due to non-specific clinical symptoms that overlap with bronchial pneumonia and lung cancer [20].

The most significant finding in this study is the detection of *G. duodenalis*, previously reported only in hedgehogs from New Zealand [23], the Netherlands [24], France [25], and Austria [26], but never before documented in Italian hedgehogs. Isolates from other countries were attributed to assemblage A [24] or to sub-assemblage AI [25], posing interesting implications from epidemiological and public health standpoints.

While feline-adapted assemblage F and canine-adapted assemblages C and D have been sporadically reported to infect humans, the presence of a zoonotic assemblage in hedgehogs could facilitate parasite transmission from wildlife to humans, either directly or through companion animals that act as bridging hosts, and vice-versa. Plausible transmission scenarios see fecal contamination of gardens and parks by infected pets or wildlife that visit human settlement for food and water. The transmission of zoonotic assemblages of *G. duodenalis* can therfore occur not just from the sylvatic to the domestic environment, but also the other way around, with infected pets transmitting the parasite to wildlife that can then spread it in the environment.

This case therefore highlights the importance of passive surveillance efforts carried out to detect zoonotic pathogens in wild animals, both to understand transmission pathways and to prevent zoonotic transmission. Further molecular studies, implementing the protocol with MLST genotyping should be extensively carried out to ascertain with more precision which are the *G. duodenalis* assemblages circulating in Italian wildlife.

Understanding the potential risks associated with wildlife encroachment into urban environments is crucial and effective strategies are needed to promote coexistence in areas where urban and natural environments overlap. It is essential to discourage wildlife habituation to human presence and to reduce the availability of food sources that attract wild animals and their pathogens. Additionally, attention should be directed towards individuals engaging closely with wildlife such as researchers, veterinarians, rehabilitators, and ordinary citizens who may encounter injured or abandoned wild animals, promoting educational initiatives to clarify the health risks linked to improper wildlife handling.

## Data Availability

All data generated is available from the corresponding Author upon reasonable request. The genetic sequence produced is deposited in the GenBank international repository under the accession number PP960564.
